# Tox induces T cell IL-10 production in a BATF-dependent manner

**DOI:** 10.3389/fimmu.2023.1275423

**Published:** 2023-11-20

**Authors:** D. Alejandro Canaria, J. Alejandra Rodriguez, Luopin Wang, Franklin J. Yeo, Bingyu Yan, Mengbo Wang, Charlotte Campbell, Majid Kazemian, Matthew R. Olson

**Affiliations:** ^1^ Department of Biological Sciences, Purdue University, West Lafayette, IN, United States; ^2^ Department of Computer Science, Purdue University, West Lafayette, IN, United States; ^3^ Department of Biochemistry, Purdue University, West Lafayette, IN, United States

**Keywords:** T cell differentiation, BATF/JUN/IRF4 complex, TOX, IL-10, transcription factors (TF) S

## Abstract

Tox is a member of the high mobility group (HMG)-Box transcription factors and plays important roles in thymic T cell development. Outside of the thymus, however, Tox is also highly expressed by CD8 and CD4 T cells in various states of activation and in settings of cancer and autoimmune disease. In CD4 T cells, Tox has been primarily studied in T follicular helper (TFH) cells where it, along with Tox2, promotes TFH differentiation by regulating key TFH-associated genes and suppressing CD4 cytotoxic T cell differentiation. However, the role of Tox in other T helper (Th) cell subtypes is less clear. Here, we show that Tox is expressed in several physiologically-activated Th subtypes and its ectopic expression enhances the *in vitro* differentiation of Th2 and T regulatory (Treg) cells. Tox overexpression in unpolarized Th cells also induced the expression of several genes involved in cell activation (*Pdcd1*), cellular trafficking (*Ccl3, Ccl4, Xcl1*) and suppressing inflammation (*Il10*) across multiple Th subtypes. We found that Tox binds the regulatory regions of these genes along with the transcription factors BATF, IRF4, and JunB and that Tox-induced expression of IL-10, but not PD-1, is BATF-dependent. Based on these data, we propose a model where Tox regulates Th cell chemotactic genes involved in facilitating dendritic cell-T cell interactions and aids in the resolution or prevention of inflammation through the production of IL-10.

## Introduction

Tox is an HMG-Box domain transcription factor that plays multiple roles in immune cell development and function. Tox was initially identified as a mediator of thymic T cell expansion and the development of lymphoid inducer, innate lymphoid and natural killer cells (1–4). In the process of thymic T cell development, Tox regulates expression of the CD4 T cell lineage-directing factor ThPOK and in the absence of Tox, CD4 T cell development is impaired (1, 3). While Tox is clearly important for thymic T cell development, it is also been shown to be expressed in peripheral mature T cells in various states of activation and disease.

Outside of thymic development, Tox is also involved in the activation and function of mature CD8 T cells. Previous work indicates that while Tox is minimally expressed in resting mature T cells, it is induced after T cell receptor (TCR) signaling (5). In settings of chronic TCR stimulation that occurs during chronic viral infection or within tumors, Tox expression heightened and is elevated for a prolonged period of time. This dysregulated Tox expression acts to induce the immune checkpoint molecules (PD-1, TIGIT, LAG3) that suppresses T cell anti-viral/tumor activity and limits cytokine production. Further, Tox promotes long-term alteration of the epigenetic landscape of other key effector genes that aid in maintaining this poorly functional state (4–7). Somewhat paradoxically, Tox is also important for the persistence of CD8 T cells in settings of central nervous system inflammation and chronic antigen stimulation (8–10) suggesting that Tox plays multiple roles in regulating T cell function.

Tox expression is also elevated in a subset of highly functional (i.e. non-exhausted) CD4 T cells, termed T follicular helper (TFH) cells that specialize in providing help for the B cell response (11, 12). Here, Tox cooperates with the related Tox2 transcription factor to drive TFH cell differentiation (11, 12). Similar to CD8 T cells, Tox/Tox2 induce expression of PD-1 in CD4 T cells (11, 12), which has been previously been shown to aid in the positioning of TFH cells near or within the germinal center (13). These data indicate that Tox is a critical regulator of the differentiation, trafficking and function of CD4 TFH cells. However, the expression of Tox in other Th cell subtypes (i.e. Th1, Th2, Treg) and whether or not Tox may contribute to their lineage-specific functions is less well understood.

We show here that Tox is also expressed by physiologically-activated T-bet^+^ Th1 and Foxp3^+^ T regulatory (Treg) cells indicating that it may carry out an important function in multiple Th subtypes. Retroviral-mediated expression of Tox modestly enhanced Th2 and Treg differentiation and suppressed the differentiation of Th1 cells. Further, ectopic Tox expression in unpolarized T cells induced the transcription of several genes involved in cell migration (*Ccl3, Ccl4, Xcl1*) and in the production of the anti-inflammatory cytokine IL-10. We further show that Tox directly binds several of these gene loci with the transcription factors BATF, IRF4 and JunB and that BATF is required for Tox-induced IL-10 production, but not expression of PD-1. Together, these data suggest that Tox induces a set of chemokine genes in CD4 T cells that may be involved in recruiting immune cells to sites of inflammation and modulating inflammation through the production of IL-10.

## Materials and methods

### Mice

C57BL/6 and OT-II mice that were used in this study were originally procured from Jackson Laboratories, and *Batf^-/-^* mice which were a gift by Dr. Elizabeth Taparowsky (Purdue University, West Lafayette, IN). The mice were bred and maintained in the AAALAC-certified Purdue University Animal Housing facility as mentioned above, and the experiments used for these studies were under the protocol approved by Purdue University Institutional Animal Care and Use Committee (IACUC).

### *Ex vivo* cell isolation

Spleens, Peyer’s Patches, and lymph nodes were dissected from WT C57BL/6 mice and processed for cell isolation. For spleen and lymph nodes, the organs were collected in sterile MACS buffer and were macerated using frosted glass slides. Subsequently, the splenic and lymph node cell suspensions were treated with red blood cell lysis buffer (155mM Ammonium Chloride, 12mN Sodium Bicarbonate, 0.1mM EDTA) and resuspended in complete RPMI media (penicillin-streptomycin (Gibco),10% Fetal Bovine Serum and 50µM 2-mercaptoethanol (Gibco) for subsequent culturing or flow cytometric analyses.

### Naïve CD4 T cell isolation and culture

Spleens from WT C57BL/6 mice were processed as mentioned above to obtain naïve CD4 T cells using a magnetic isolation naïve CD4 T cell isolation kit (Miltenyi Biotec) as per manufacturer’s instructions. The purity of enriched of naïve (CD44^lo^, CD62L^hi^) CD4 T cells was > 90%. 1x10^6^ enriched cells/mL were cultured in plate-bound anti-CD3ε (2 µg/mL, 2C11, Bio X cell) pre-coated plates (in 1x DPBS (Gibco) overnight at 4°C) and complete RPMI media supplemented with soluble anti-CD28 (4 µg/mL, 37.51, Bio X cell), and the following differentiation conditions for Th cell differentiation: for unpolarized Th cells, we added recombinant human IL-2 (100 Units/mL, Peprotech). For Th1 cells, we used recombinant mouse IL-12, (10 ng/mL, Peprotech) and anti-IL-4 (12.5 µg/mL, 11B11, BioXCell). For Th2 cells, we supplemented with recombinant mouse IL-4 (20 ng/mL, Peprotech) and anti-IFNγ (12.5 µg/mL, XMG1.2, BioXcell). The Th17 cell differentiation conditions were anti-IL-4, anti-IFNγ, recombinant murine IL-6 (100 ng/mL, Peprotech), TGF-β1 (1 ng/mL, Peprotech), and anti-IL-10R (12.5 µg/mL, 1B1.3A, BioXcell), and for Treg cells we used recombinant human IL-2, TGF-β1 anti-IL-4 and anti-IFNγ. In some experiments, half cytokine levels from that indicated above were used for initial Th cell differentiation to mimic suboptimal differentiation conditions. After plating, the cells were incubated for 4 days at 37°C in a 5% CO2 incubator and rested (no anti-CD3 or anti-CD28) for 1 day in 3 volumes of media with half the amount of cytokines as used in the initial culture conditions. Afterward, the cells were harvested and processed for flow cytometry.

### Total lymph node cell cultures

We isolated mesenteric and inguinal lymph nodes from *Batf^+/+^* and *Batf^-/-^* mice. Subsequently, we crushed the lymph nodes using frosted glass slides to generate a single-cell suspension that was filtered through a 0.35 μm cell strainer, to eliminate debris. The cell suspension was counted with a hemocytometer and T cell frequencies in each preparation were determined by flow cytometry. We then cultured 2X10^6^ lymphocytes/mL of complete RPMI media in anti-CD3 coated plates with anti-CD28 and recombinant human IL-2 (100 U/mL) for 2 days before transduction, expanded as above and harvested on day 5 for flow cytometric analysis.

### Retroviral generation and transduction

We generated the retrovirus as described previously (14, 15). Briefly, we transfected HEK-293T with 20 μg MSCV-IRES-eGFP (empty vector control, a gift from Dr. Mark Kaplan at Indiana University School of Medicine) or MSCV-Tox-IRES-eGFP and 10 μg of the packaging vector pCL-Eco using Lipofectamine 2000 (Invitrogen) as per manufacturer’s instruction. 48 hours after transfection, we collected the supernatant, centrifuged for 10min at 4°C at 500xg, to remove any remaining HEK-293Tcells, and transferred this cleared supernatant to new tubes for subsequent transduction. For transduction, 2 days after T cell activation with anti-CD3 and anti-CD28 (as per above), we centrifuged the plates containing the T cell cultures for 5 min at 30°C at 300 g before removing and saving the T cell media from each well. We then added viral supernatants to spin-infect the T cells for 90 min at 30°C at 500xg in the presence of 8 μg/mL of polybrene. After centrifugation, the viral supernatant was removed and discarded, and the saved T cell media was added back into the wells. Cells were expanded on day 4 as per above and harvested on day 5. The efficiency of transduction was determined by assessing GFP or Thy1.1 expression through flow cytometry.

### Flow cytometry, intracellular cytokine and transcription factor staining

Cultured cells were harvested and ~2x10^5^ cells at 10^6^ cells/mL were used for either cytokine staining (ICS) or transcription factor (TF) staining. For ICS, the Th cells were incubated at 37°C with PMA (0.5 μg/mL) and ionomycin (0.5 μg/mL, Sigma) for 2.5 hours and monensin (2 μM) (Biolegend) was added for additional 2.5 hours. Subsequently, these cells were stained with a fixable viability dye (ef780, Thermo Fisher) and mouse anti-CD4 (BV510, RM4-5, Biolegend) or anti-CD8a antibodies (BV605, 53.6-7, Biolegend) for 20 min at 4°C were then washed with FACs buffer followed by fixation with 3.7% formaldehyde (15 min at room temperature). After fixation, cells were permeabilized with Intracellular Staining Perm Wash Buffer (Biolegend) and stained in the same buffer with a cytokine antibody cocktail including antibodies against mouse IL-4 (PE-Cy7, 11B11, Biolegend), IL-5 (APC, TRFK5, Biolegend), IL-13 (Percp-Cy5.5, W17010B, Biolegend), IFN-γ (FITC/Percp-Cy5.5, XMG1, Biolegend), IL-10 (PE/FITC, JES5-16E3, Biolegend), CCL3 (PE, DNT3CC, Thermo Fisher) and CCL4 (AbByFlour647, polyclonal, Bioss). For TF staining, the cells were first stained with cell surfaces markers as above and fixed for 15 min (room temperature) with 1.5% formaldehyde followed by a secondary fixation with True-nuclear fixation kit (Biolegend) according to the manufacturer’s instructions. After this secondary fixation, the cells were permeabilized with True-nuclearTM Perm buffer (Biolegend) and were stained with antibodies to TOX (ef660, TXRX10, Thermo Fisher), T-bet (PE/Percp-Cy5.5, 4B10, Biolegend), Rorγt (PE/AF660, B2D, Thermo Fisher), GATA3 (PE, TWAJ, Thermo Fisher) and FOXP3 (AF488/PE, FJK-13s, Thermo Fisher). After washing 2 times with FACs buffer, stained samples were run on an Attune NxT flow cytometer (Thermo Fisher), and the data were analyzed using FlowJo software (v.10.0).

In other experiments, we performed phenotyping of Peyer’s Patch (PP) CD4 T cells. For these studies, single cell suspensions of PP cells were stained with a fixable viability dye (as above) and purified anti-mouse CD16/32 (Biolegend) for 20 min in PBS. These cells were then stained with cell surface CD4, CD8, CXCR5 (PE/Cy7, L138D7, Biolegend), PD-1 (PE-Dazzle594/Percp-Cy5.5, RMP1-30, Biolegend) antibodies followed by intranuclear staining for T-bet and FOXP3 as above. Cells from these studies were also run on an Attune NxT flow cytometer and the data were analyzed using FlowJo software.

### RNA isolation and RNA-seq analyses

For RNA-seq studies, we isolated naïve CD4 T cells as explained above and cultured them under unpolarized conditions using plate bound anti-CD3, soluble anti-CD28 and recombinant human IL-2 as above. After 2 days in culture, we transduced the cells with retroviruses encoding Tox or the respective empty vector control as per above. Two biological replicates per condition were used. The cells were expanded as explained above and on day 5, we stimulated the cells with biotinylated anti-CD3 and streptavidin for 5 hours (6). After stimulation, we sorted CD4 GFP^+^ cells to enrich positively transduced T cells. The resulting cells were washed twice with 1x sterile PBS the pellets were resuspended in 500 μL of Trizol reagent (Applied Biosystems) and RNA-isolation was carried out as per the manufacturer’s instructions. RNA from these cells was sent to Novogene for library preparation and sequencing. For RNA-seq analyses, the raw sequencing quality was checked using fastqc. Alignment and RNA expression level calculation was carried out using bowtie1 and RSEM (–bowtie-n 1 –bowtie-m 100). The reference transcript used in alignment was mm10. DESeq2 was used to identify the differential expressed genes between CD4 T cells transduced with Tox retrovirus and with Empty Vector retrovirus with default parameters. Differentially expressed genes (DEGs) were sorted based on significance level [log2(fold change)*-log2(p-value)]. All packages involved in the downstream analyses are available as R packages. RNA-seq data for this project have be deposited in the NCBI gene expression omnibus (GEO) under GSE240348.

### ChIP-seq and CUT&RUN data analyses

Processed data of CD8 BATF, IRF4 and JunB ChIP-seq data were sourced from GSE111902. Raw sequencing CUT&RUN data of CD8 IgG and Tox were downloaded from GSE175437 (16); CD4 BATF, IRF4 and JunB ChIP-seq data was obtained from GSE172490 (17). Sequenced reads were aligned to mm10 using Bowtie (18) with parameters “–chunkmbs 1000 -S -p 10 -m 1”. The bam files were indexed using samtools 1.17 (19). Peaks were called using callpeak function from macs2 2.1.4 (20) with parameter “–nomodel”, which were further filtered against mm10 blacklist regions downloaded from https://github.com/Boyle-Lab/Blacklist/blob/master/lists/mm10-blacklist.v2.bed.gz. To annotate the peaks to nearest genes, annotatePeaks.pl (21) was utilized with default parameters. Bigwig files were generated using deepTools2 (22) with parameters “bamCoverage –normalizeUsing BPM –minMappingQuality 30 –ignoreDuplicates –blackListFileName mm10-blacklist.v2.bed”. Tracks were visualized through Integrative Genomics Viewer (IGV, Web App) (23).

### Quantitative RT-PCR

Gene expression analysis was carried out using real-time quantitative PCR. We synthesized cDNA using 1 μg of RNA from cultured cells or sorted GFP^+^ CD4 T cells transduced with a Tox retrovirus or an Empty Vector retrovirus using the High-Capacity cDNA Reverse Transcription Kit (Thermo Fisher Scientific). Quantitative PCR was done using theTaqMAN™ Fast Advanced Master Mix (Thermo Fisher Scientific) and the probes: *Tox* (Mm00455231_m1)*, Pdcd1*(Mm01285676_m1), and *Il10* (Mm01288386_m1) from Thermo Fisher Scientific, and *Xcl1*(Mm.PT.58.10915849) and *Ccl3*(Mm.PT.58.29283216) from Integrated DNA Technologies (IDT). Obtained ct values were used for assessing relative expression analysis using the 2^-ΔΔct^ method.

## Results

### Tox is expressed in multiple physiologically-activated Th cell subtypes

**Figure 1:**
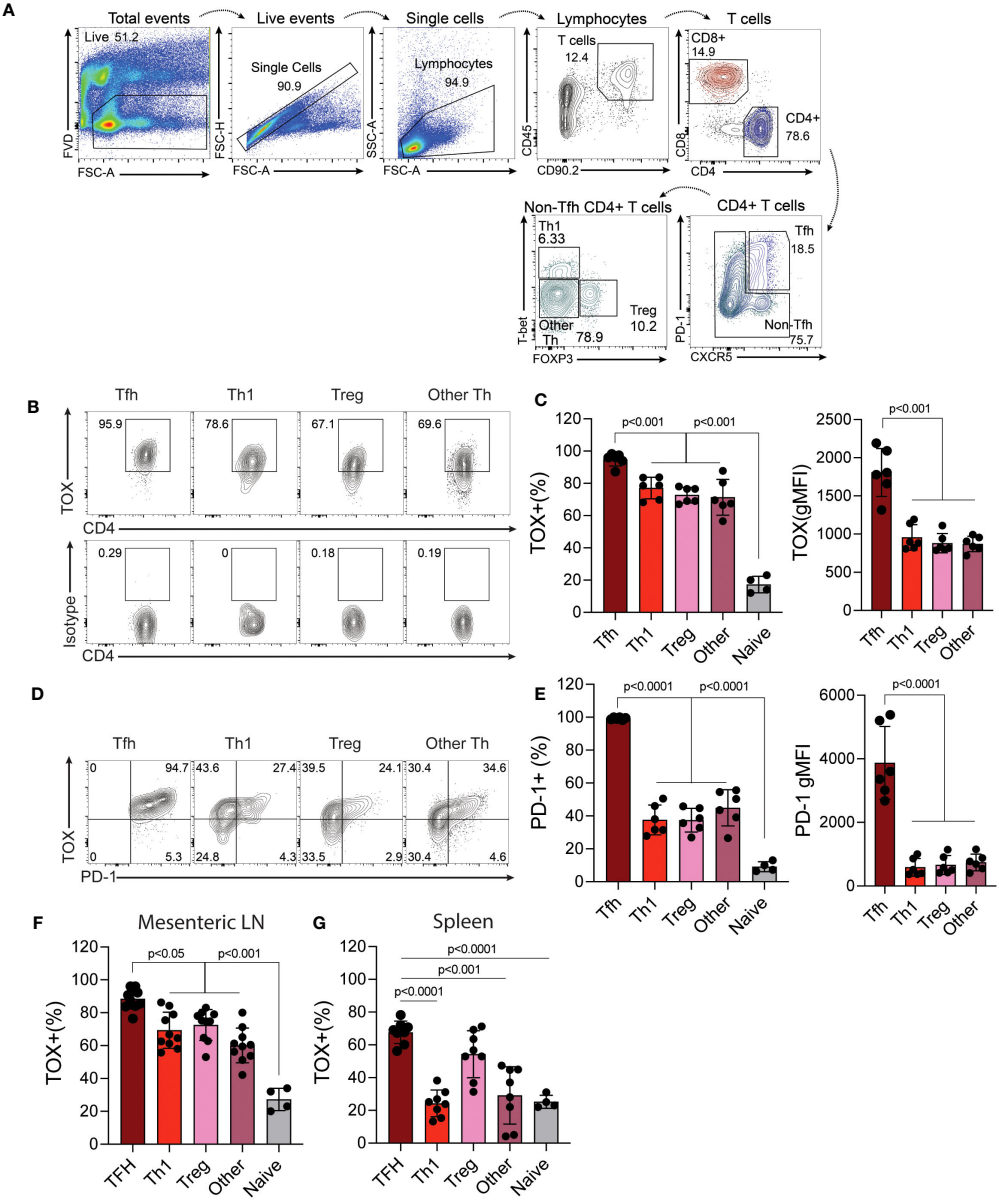
Tox is expressed by multiple *in vivo*-derived Th cell subtypes. Peyer’s patches (PP) were harvested from C56BL/6 WT healthy 8-12-week-old mice and the single cell suspensions were analyzed to examine Tox expression in CD4 T cells. **(A)** Gating strategy: Viable cells were selected as fixable viability dye (FVD) negative, single cells by linear forward scatter (FSC) area to height ratio, lymphocytes were gated based on FSC and side scatter (SSC) properties, T cells were identified as CD45^+^ and CD90.2^+^, CD4 T cells were identified as CD4^+^, TFH were selected as CXCR5^+^ and PD-1^+^. CXCR5^-^/PD-1^-^ cells were selected for identification of Th1 cells- T-bet^+^, Tregs- Foxp3^+^ and Th “other”- T-bet^-^/Foxp3^-^. Naïve CD4 T cells were identified as CD44^lo^ and CD62L^+^ (gating not shown). **(B)** Representative contour plots of Tox and isotype staining in the indicated populations. **(C)** Quantified Tox frequencies and geometric mean fluorescence intensity (gMFI). **(D)** Representative contour plots of Tox and PD-1 expression of indicated Th cell populations. **(E)** Quantified frequencies and gMFI of cell surface PD-1 protein expression in each Th cell subset. **(F)** % of Tox-expressing CD4 T cells of each subtype in the Mesenteric lymph node (LN) and spleen **(G)**. MFIs for naïve CD4 T cells are not shown as they were analyzed using different instrument settings as compared to effector Th cell populations. Positive staining for Tox in naive cells and effector T cells was based on an isotype control that was run with these samples. Data was considered significant when P-values were <0.05 in a multiple comparison ordinary one-way ANOVA test. n.s., non-significant; n.d., not done. These data are representative of cells isolated from 6-10 individual mice from 2-3 individual experiments.

Previous work indicates that Tox is highly expressed by Th cells in tumors and those within or surrounding germinal centers (termed T follicular helper cells). However, whether or not Tox is expressed by other Th subtypes outside of these environments is less clear. To address this, we isolated cells from the mouse Peyer’s patches (PP), mesenteric lymph nodes (MLN) as a source of physiologically-activated Th cells and the spleen as a location that has relatively fewer activated T cells and is not associated with the intestinal tract. From these cells, we analyzed the expression of Tox in different Th subtypes (TFH; CXCR5^+^ PD-1^+^, Th1; CXCR5^-^, PD-1^-^, T-bet^+^, Treg; CXCR5^-^, PD-1^-^, Foxp3^+^, Th2; CXCR5^-^, PD-1^-^, GATA3^+^) using the gating strategy in **Figure 1A**. It should be noted that the data below were recapitulated using antibodies to CD3 as an indicator of T cell identity, in place of Thy1, and this resulted in virtually identical results (data not shown). As expected, virtually all TFH cells in the PP, MLN and spleen expressed high levels of Tox which [frequency and mean fluorescence intensity (MFI)] as compared to all other Th subtypes examined (**Figures 1B, C, F, G**). Despite being lower than TFH cells, we observed that ~75% of Th1, Tregs and other undefined Th cells within the PP and MLN also expressed Tox above what was seen in naïve CD4 T cells (CD44^lo^, CD62L^+^) indicating that Tox may contribute to the differentiation and/or function of effector Th cells in this tissue (**Figures 1B, C**). However, the frequency of Tox^+^ Th1 and undefined Th cells were dramatically reduced to naïve CD4 T cell levels in the spleen (**Figure 1G**) while a Tregs maintained their Tox^+^ status in this organ. These data suggest that Tox may be regulated by distinct mechanisms in Th cell subtypes from different tissues. For Th1 and Th “other” cells, Tox may be regulated by gut-specific activation signals or inflammatory cytokines. Indeed, Tox has been shown to be induced by gut-specific signals and inflammatory cytokines in intestinal T cells (24, 25). Further, Tox has been indicated as a general marker of activated T cells in humans (10) which is consistent with the notion that intestinal T cells are constantly activated by signals from the gut microbiota. For TFH and Tregs, it is possible that Tox is induced by signals required for the differentiation of these cell types. As mentioned above, Tox promotes TFH differentiation (11, 12) and has been previously described in Treg cells (26). Unfortunately, we did not detect large enough numbers of GATA3^+^ Th2 cells in these tissues to reliably quantify Tox in this cell type (<0.5% GATA3^+^ cells present, **Figures 1B, C**) and were unable to examine Rorγt expression in this staining panel.

Previous work indicated that Tox regulates the expression of PD-1 in CD8 T and TFH cells (4–7, 11, 12, 27). We therefore asked if Tox also correlated with PD-1 expression in these other Th subtypes. Interestingly, we found that while virtually all TFH cells co-expressed PD-1 and Tox to some degree and (e.g. virtually all PD-1^+^ cells were Tox^+^), PD-1 expression was reduced in Th1 and Th “other” cells, with a substantial population of Tox^+^ cells that did not co-express PD-1 (**Figures 1D, E**). As mentioned above, this disconnect in Tox and PD-1 expression may be due to differences in inflammatory signals or the activation state of these cells in each tissue (10, 24, 25). Taken together, these data show that Tox is highly expressed in physiologically-activated Th cells across multiple tissues and may contribute to functions beyond PD-1 expression and functional exhaustion.

### Ectopic Tox expression marginally alters Th cell differentiation

**Figure 2:**
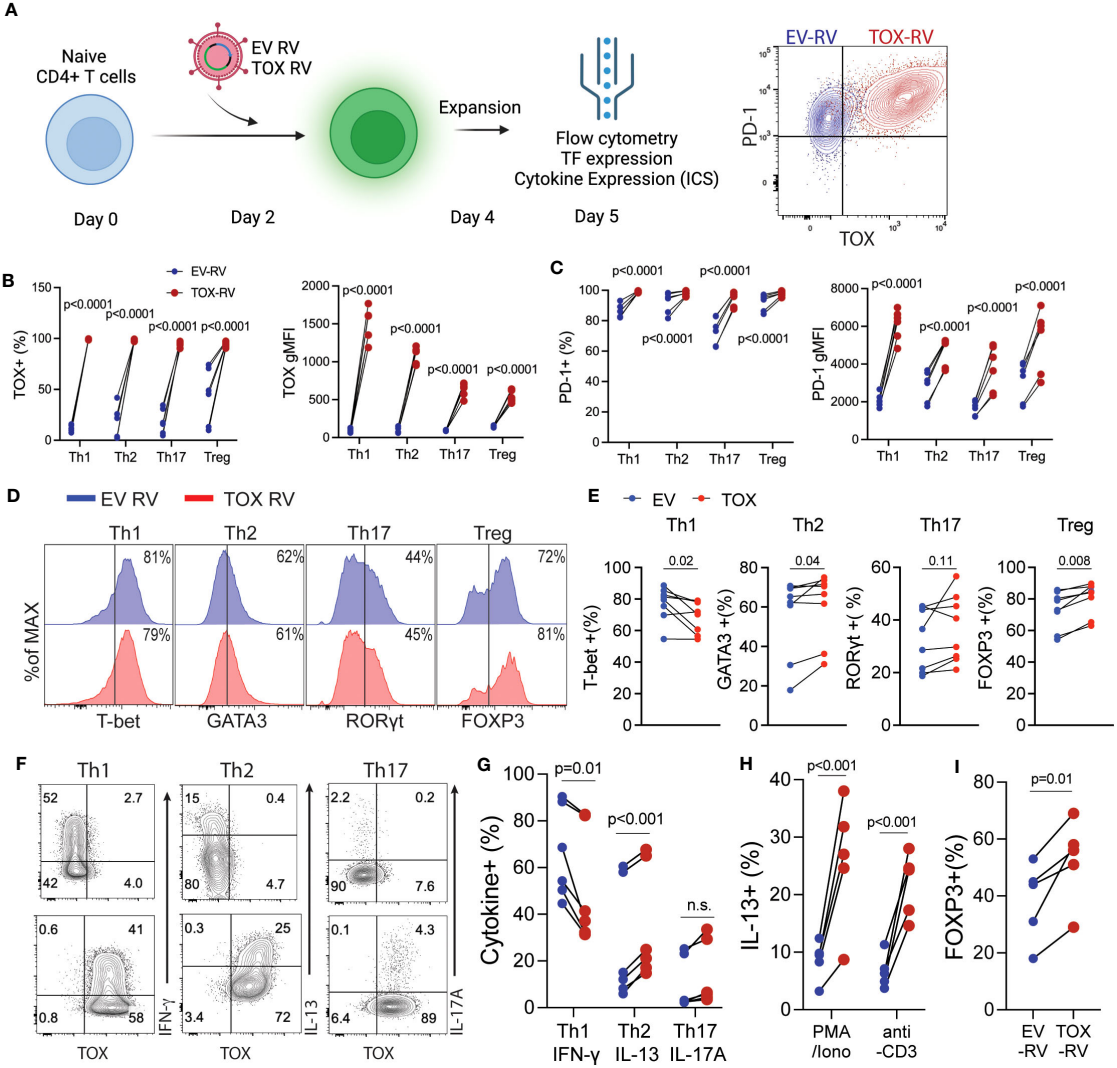
Elevated Tox expression modestly affects CD4 T helper cell differentiation *in vitro*. **(A)** Schematic of the experimental procedure: Naïve CD4 T cells were activated *in vitro* under Th1, Th2, Th17 or Treg conditions for 2 days followed by transduction with a GFP-expressing empty retrovirus (EV RV) or a GFP/Tox-expressing retrovirus (Tox RV). Resulting cells were harvested on day 5 of culture. Right panel, representative contour plot indicating PD-1 and TOX expression from EV-RV (blue) or TOX-RV-transduced (red) Th1 cells. **(B)** The % and gMFI of Tox^+^ GFP^+^ CD4 T cells in each condition. **(C)** The % and gMFI of PD-1^+^ GFP^+^ CD4 T cells in each condition. Data are representative of cultures performed from 5-6 mice from 3 individual experiments. **(D)** Representative histograms of Th cell-defining transcription factors in the indicated GFP^+^ Th cell subtype. **(E)** Quantification of the % of transcription factor^+^ CD4 T cells in EV RV- or Tox RV-transduced T cells (GFP^+^). Data are representative of cultures performed from 6-8 mice from 3 individual experiments. **(F)** At day 5 of culture cells were stimulated with PMA and ionomycin and the frequency of cytokine^+^ of GFP^+^ T cells was determined by flow cytometry. Representative contour plots of cytokine ICS are shown for Th1, Th2 and Th17 cells. **(G)** Quantification of cytokine production in each Th cell subtype. Data are representative of cultures performed from 6 mice across 3 individual experiments. n.s., not significant. **(H)** IL-13 production from Th2 cells cultured with half of the polarizing cytokine production (i.e. suboptimal Th2 conditions). Resulting cells were stimulated with either PMA/Ionomycin or anti-CD3 to elicit cytokine production for intracellular staining. **(I)** % of FOXP3^+^ cells from cells cultured under half (i.e. suboptimal) Treg polarizing cytokine conditions. Data are representative of cultures from 5 mice across 2 individual experiments. Data was considered significant when P-values were <0.05 in a paired Students t-test.

Based on our findings above showing that Tox was expressed in *in vivo*-derived Th cells, we hypothesized that Tox may contribute to Th cell differentiation. To test this hypothesis, we *in vitro* differentiated Th1, Th2, Th17 and Treg cells for 2 days and transduced them with a GFP-expressing empty vector (EV) or a Tox-GFP retrovirus (Tox RV). After transduction, cells were cultured for an additional 2 days and then rested for 24 hours in a fresh media without TCR stimuli. Resulting GFP^+^ cells were then assessed for Tox/PD-1 expression, Th cell lineage-specific transcription factors (Th1; T-bet, Th2; GATA3, Th17; Rorγt, Treg; Foxp3) and cytokines (Th1; IFN-γ, Th2; IL-4/IL-13, Th17; IL-17A) by flow cytometry (**Figure 2A**). In contrast to *in vivo*-derived cells, *in vitro*-generated Th1, Th2, Th17 and Tregs expressed very little Tox protein, however, this was markedly elevated in GFP^+^ cells from all Th subtypes that were transduced with Tox RV as compared to EV-transduced cells (**Figures 2A, B**). To ensure that ectopic Tox transduction was functional in each cell type, we also examined PD-1 expression (a known Tox target gene) on GFP^+^ cells from EV RV or Tox RV-transduced cells. Independent of Th cell type, all cells upregulated PD-1 when Tox was ectopically expressed as compared to EV RV controls (**Figures 2A, C**). Despite Tox’s ability to drive PD-1 in these conditions, it marginally impacted Th cell differentiation. Ectopic Tox expression modestly decreased levels of the Th1 factors T-bet and IFN-γ and slightly upregulated Th2 GATA3 expression. Despite the minimal increase in GATA3 expression, Tox-transduced Th2 cells exhibited an enhanced capacity to produce the Th2 cytokine IL-13 (**Figures 2F, G**). As Tox-induced factors may interfere with T cell receptor (TCR) signaling, we questioned whether or not Tox would still induce IL-13 production when stimulated through the TCR. To this end, we stimulated EV- and Tox-transduced Th2 cells with PMA/Ionomycin (TCR-independent stimulus) or anti-CD3 (TCR-dependent stimulus) and measured intracellular IL-13 production by flow cytometry (note that Th2 cells used for these experiments were cultured under suboptimal Th2 conditions- 5ng/ml IL-4, to better observe Tox-induced IL-13 production). We observed that Tox-enhanced IL-13 production in both PMA/Ionomycin and anti-CD3 conditions indicating that Tox unlikely interferes with T cell receptor signaling in inducing Th2 cytokine production (**Figure 2H**). In contrast to Th2 cells, we observed no changes in the Th17-associated factor Rorγt and a modest increase in the frequency of IL-17-producing cells when cultured under basal Th17 polarizing conditions (TGF-β and IL-6 only) (**Figures 2D–G**). Under optimal Treg conditions, we also observed a modest increase in Foxp3 expression (**Figures 2D, E**), and this increase in FOXP3 was further exaggerated when we used suboptimal Treg culturing conditions (1/2 amounts of IL-2 and TGF-β as compared to optimal conditions) (**Figure 2I**). These data suggest that Tox may play a role in enhancing Treg and Th2 polarization under suboptimal conditions, but is not likely to be a major Th cell lineage-driving transcription factor.

### Tox regulates genes associated with cell migration and IL-10 production in CD4 T cells

**Figure 3:**
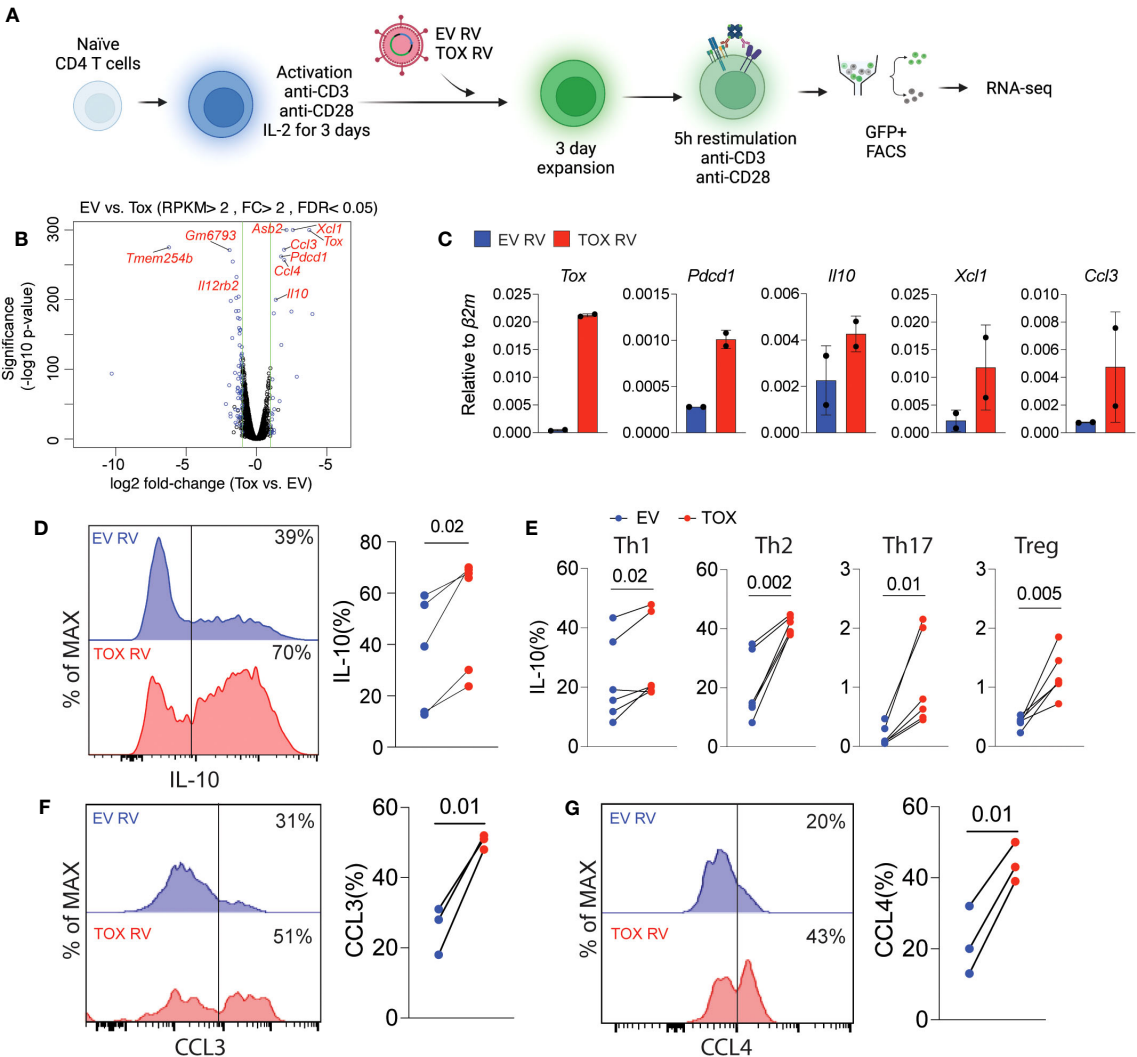
Ectopic Tox expression induces transcription of chemotactic genes and the anti-inflammatory cytokine IL-10. **(A)** Schematic of experimental procedure of Tox overexpression in unpolarized CD4 T cells, retroviral (RV) transduction, cell sorting and RNA-seq. **(B)** Volcano plot of differentially expressed genes in GFP^+^ sorted Tox vs EV overexpressing CD4 T cells (each group represents 2 individual mice). Significantly different genes are highlighted. **(C)** qRT-PCR validation of selected Tox-induced genes from 2 individual mice. **(D)** Representative histograms and quantification of IL-10 in transduced (GFP^+^) T cells from cultures from 4 individual mice. **(E)** The % of IL-10 producing T cells of GFP^+^ Th1, Th2, Th17 and Treg cultures cells transduced with EV RV or Tox RV. Cultures were from 5-6 mice in 2 independent experiments. **(F, G)** The % of CCL3^+^ and CCL4^+^, respectively, of Th0 condition cultured CD4 T cells that were obtained from lymph nodes. These data are representative of cells cultured from 3 mice from 2 independent experiments. Data was considered significant when P-values were <0.05 in a paired Students t-test. N.

Based on these relatively modest changes in Th cell differentiation, we questioned what other genes or cellular pathways may be regulated by Tox in Th cells. To this end, we transduced naïve CD4 T cells that had been activated for 2 days with EV RV or Tox RV under non-polarizing (i.e. anti-CD3, anti-CD28, and IL-2 only) conditions. Anti-CD3 stimulation was maintained for an additional day after transduction, followed by 2 days of “rest” in a non-anti-CD3-coated plate. After a total of 5 days of culture, we re-stimulated the cells using anti-CD3 and anti-CD28 as previously reported (6), sorted the GFP^+^ cells and performed RNA-seq on RNAs isolated from these cells (**Figure 3A**). We found that ectopic Tox expression led to statistically differential expression of a relatively small set of 22 genes, including 11 upregulated and 11 downregulated genes (fold change >2; FDR <0.05), as compared to previous work in CD8 T cells where hundreds of genes were modulated by ectopic Tox expression (4, 6). Expectedly, both *Tox* and its known downstream target gene *Pdcd1* were among the top upregulated genes in this data set (**Figure 3B**). Further, we found that Tox downregulated the expression of a component of the IL-12 receptor (*Il12rb)*, which is important for Th1 cell differentiation (28). This matches the modestly reduced Th1 T-bet and IFN-γ expression observed above (**Figures 2D–F**).

Remarkably, we found that ectopic Tox expression was associated with an increase in chemokine genes involved in cell migration such as *Xcl1*, *Ccl3* and *Ccl4* (**Figure 3B**). In addition, we also found that Tox overexpression resulted in increased expression of the anti-inflammatory and Th2/Treg-associated cytokine IL-10. This corresponds with our data above showing that Th2 Treg differentiation were also modestly induced by Tox (**Figures 2D–F**). To verify these RNA-seq findings, we repeated these retroviral transduction experiments and validated increased mRNA levels of *Tox, Pdcd1, Il10, Xcl1 and Ccl3* by qPCR from sorted GFP^+^ cells from two additional cultures (**Figure 3C**). Further, we validated increased IL-10 protein levels by intracellular cytokine staining after PMA and ionomycin stimulation of EV RV or Tox RV-transduced cells and found that IL-10 protein was significantly elevated by ectopic Tox expression (**Figure 3D**). Since we had observed IL-10 induction in non-polarizing condition, we asked if Tox promoted IL-10 production in different Th cell subtypes. Here naïve CD4 T cells were cultured under Th1, Th2, Th17 and Treg conditions and transduced with EV RV or Tox RV as above. On day 5 of culture, cells were stimulated with PMA and ionomycin and IL-10 production was measured using intracellular cytokine staining. Similar to our previous results in unpolarized cells, ectopic Tox expression led to enhanced IL-10 producing capacity in all Th cell types assessed, even modestly in Th17 and Tregs that normally make very little IL-10 *in vitro* (**Figure 3E**). We also noted that CCL3 and CCL4 chemokines were also induced by ectopic Tox expression. We validated these findings at the protein level by transducing total lymph node T cells (under non-polarizing conditions) with EV RV or Tox RV and assessed intracellular CCL3 and CCL4 protein production of GFP^+^ CD4^+^ T cells after PMA/ionomycin stimulation by flow cytometry. As predicted by our RNA-seq data, both CCL3 and CCL4 protein levels were significantly increased in Tox-transduced CD4 T cells from these cultures as compared to controls (**Figures 3F, G**).

### Tox and BATF cooperatively regulate IL-10 production in T cells

**Figure 4:**
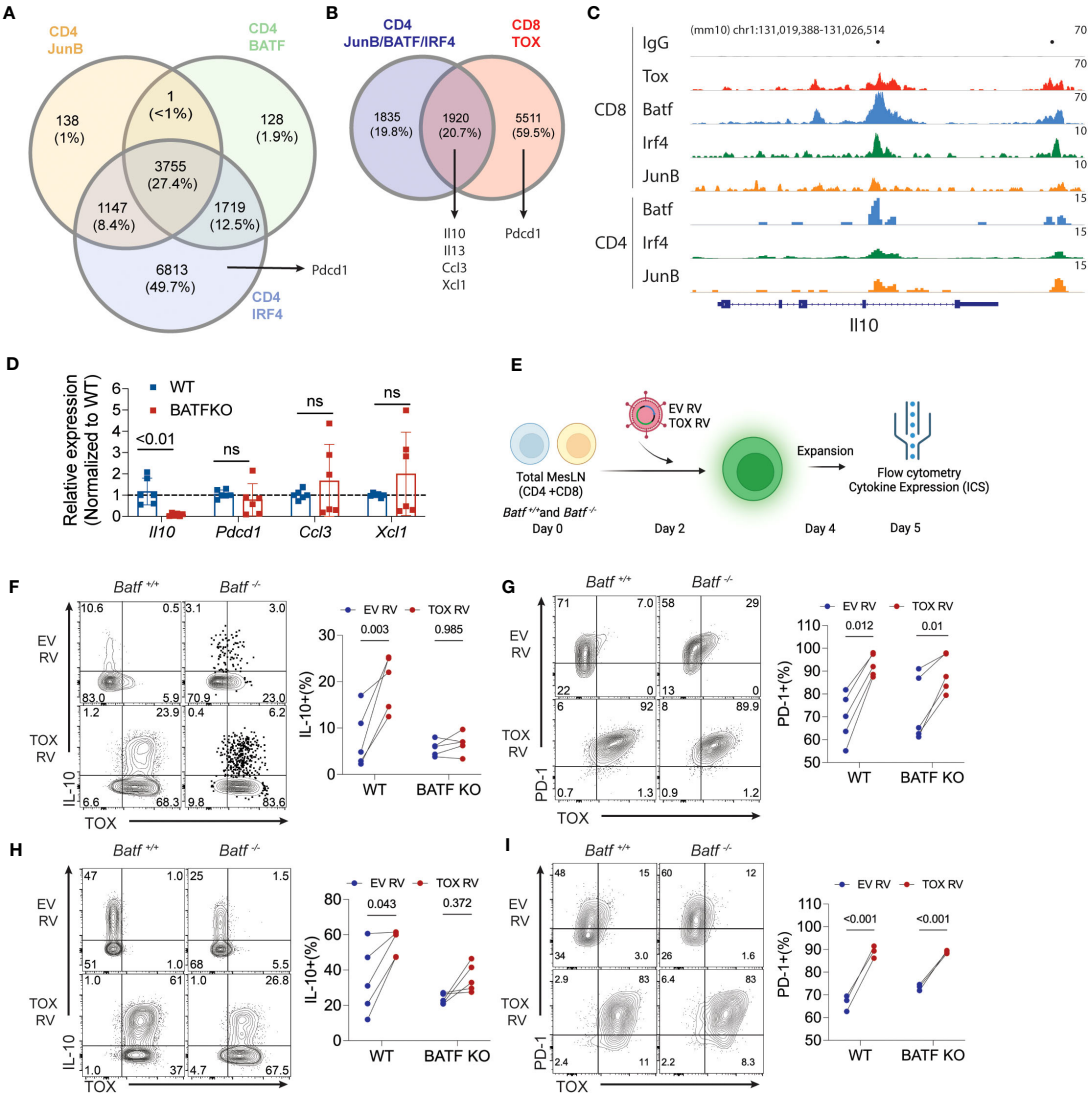
BATF is required for Tox-induced expression of IL-10. **(A)** The intersection of JunB, BATF, and IRF4 bound loci in cultured CD4 T cells. Data are sourced from GSE172490. Total number of peaks and the percentages for each intersection are shown. **(B)** The overlap between JunB/BATF/IRF4 co-bound regions from **(A)** and Tox binding loci from chronically activated/exhausted (PD1^hi^ Tim3^+^) CD8 T cells from GSE175437. **(C)** IGV genome browser tracks for IgG control, Tox, BATF and IRF4 binding to the *Il10* locus in CD8 and CD4 T cells. CD8 T cell BATF, IRF4 and JunB ChIP-seq data were sourced from GSE111902. Dots above track represent identified significantly enriched peak centers. **(D)** Real-time PCR of *Il10, Pdcd1, Ccl3, Xcl1* in 5-day cultured Th0 cells from WT or *Batf* ^-/-^ (BATF KO) mice. n.s. = not significant. Data are normalized to the median WT value for each gene represents values of cultures from 6 mice done in 2 individual experiments. **(E)** Schematic of experimental design for ectopic expression of Tox in WT or BATF-deficient total lymph node cultures. **(F, H)** Representative contour plots of Tox and IL-10 protein expression from GFP^+^ WT and BATF-deficient CD4 and CD8 T cells and quantification of these data, respectively. **(G, I)** Representative contour plots of Tox and PD-1 protein expression from GFP^+^ WT and BATF-deficient CD4 and CD8 T cells and quantification of these data, respectively. These data are representative of cultures from 4-5 individual mice from two individual experiments. P-value <0.05 was considered significant in a paired Students t-test.

Our work above indicates that Tox promotes the expression of IL-10 and PD-1 gene and protein production. However, the molecular mechanisms by which Tox drives gene expression is not well understood. A recent study in chronically-activated CD8 T cells suggested that Tox may preferentially bind AICE (AP-1-Interferon-Composite Elements) motifs in DNA and that are occupied by the AICE-binding transcription factor BATF (16). This may imply that Tox and other BATF-associated transcription factors (IRF4, JunB) cooperate to drive gene expression. To explore this possibility, we mined publicly-available BATF, IRF4 and JunB ChIP-seq data sets in cultured CD4 T cells (GSE172490) and identified loci that were bound by JunB, BATF and IRF4. As previously reported (29, 30), a large fraction of these genes (~27%) were co-bound by these three TFs in CD4 T cells, but *Pdcd1* was only bound by IRF4 (**Figure 4A**). We then examined the overlap between the loci bound by all three JunB, BATF, and IRF4 and regions bound by Tox in chronically-activated CD8 T cells (GSE175437, **Figure 4B**). Interestingly, over 51% of JunB/BATF/IRF4-binding sites in CD4 T cells are shared with Tox-bound regions in CD8 T cells. This included a number of genes that we identified in our RNA-seq analysis or were found to be induced by Tox after Th2 cell differentiation (*Il10, Il13, Ccl3, Xcl1*) (**Figure 4B**), indicating that these genes may be co-regulated by Tox and the JunB/BATF/IRF4 complex. To examine this more closely, we examined binding of these TFs at the *Il10* locus and found two distinct regions (one within the *Il10* gene body and one downstream of the *Il10* locus) that were bound by all 4 of these TFs in CD8 T cells which corresponded to JunB/BATF/IRF4 peaks in CD4 T cells (**Figure 4C**). Together, these data suggest that Tox and the JunB/BATF/IRF4 complex may specifically co-regulate IL-10 and potentially a set of genes involved in chemotactic activity in T cells.

Based on the data above, we reasoned that BATF would be required for Tox-augmented production of IL-10, but not PD-1. To initially test this possibility, we measured the expression of the Tox-induced genes that we identified in **Figures 3** and **4A, B** (*Il10, Pdcd1, Ccl3, Ccl4, Xcl1*) in Th0 cells cultured from WT and BATF-deficient mice. Consistent with a high degree of overlap between Tox and BATF binding at the *Il10* locus in CD8 and CD4 T cells, we observed a significant decrease in *Il10* expression in BATF-deficient CD4 T cells cultured under non-polarizing conditions. Also as predicted, *Pdcd1* expression was not altered in the absence of BATF (**Figure 4D**). However, we also failed to observe changes in expression of *Ccl3* and *Xcl1* expression that were predicted to also be BATF-dependent (**Figure 4D**), suggesting that other TFs may regulate *Ccl3* and *Xcl1* under non-polarized Th cell conditions. To directly test if BATF was in fact required for Tox-augmented IL-10 production, we transduced total lymphocytes from pooled mesenteric and inguinal lymph nodes (i.e. enriched CD4 and CD8 T cell population) collected from WT or *Batf* ^-/-^ mice with EV RV and Tox RV as above. At 5 days of culture, we collected cells and assessed the capacity of transduced cells (i.e. GFP^+^) to produce IL-10 and PD-1 as an additional control (**Figure 4E**). Ectopic Tox expression enhanced IL-10 protein production in both CD4 and CD8 T cells isolated from WT mice, but not from BATF-deficient animals (**Figures 4F, H**). In contrast, ectopic Tox expression induced PD-1 to a similar extent and to similar levels in both WT and BATF-deficient CD4 and CD8 T cells (**Figures 4G, I**). It is worth noting that Tox transduction did not induce enhanced BATF expression in any of the conditions that we examined (data not shown), suggesting that Tox does not simply function to enhance BATF which then induces IL-10. Taken together, these data indicate that Tox works with BATF, and possibly JunB/IRF4, to promote IL-10 production, but can also induce genes via BATF-independent (e.g. PD-1) mechanisms.

## Discussion

Tox has been previously associated with T cell exhaustion in CD8 T cells and the differentiation and function of CD4 TFH cells (4–7, 11, 12, 27). In our work, we extend upon these findings and show that Tox is also involved in promoting the production of chemokines that aid in T cell-myeloid cell interactions and in the production of the immunoregulatory cytokine IL-10. Further, we provide a molecular mechanism by which Tox and BATF, IRF4 and JunB co-occupy these loci and that Tox-induced production of IL-10 is BATF-dependent. These findings are among the first to define a role for Tox outside of CD8 T cell function/exhaustion or TFH differentiation and how Tox works with other TFs to drive gene expression.

Tox has distinct functions in different T cell types. Elevated Tox expression in chronically-activated CD8 T cells leads to functional exhaustion and a reduced capacity to drive tumor clearance via induction of cell surface inhibitory proteins like PD-1 and epigenetic modification of these cells (4–7, 27). In CD4 T cells, Tox and Tox2 cooperate to drive TFH differentiation where it induces PD-1 expression, but also other TFH-specific genes (i.e. *Bcl6, Il6st, Batf*) (11, 12, 31). In our work, we show that Tox is not only expressed by TFH cells, but it is also expressed by other Th cell types (i.e. Th1, Tregs) within the gut-associated Peyer’s Patch and lymph nodes (**Figure 1**). It is of note that Tox is also expressed by gut intraepithelial T cells (24) and these data combined with our study suggests that there may be gut-specific factors that promote Tox expression outside of chronic TCR stimulus. Functionally, we also showed that Tox modestly suppressed Th1 cell differentiation and increased Th2, Th17 and Treg differentiation and/or cytokine production. This may be due to Tox-induced repression of *Il12rb2* (**Figure 3B**), a gene encoding a chain of the IL-12 receptor that is required for IL-12 signaling and Th1 differentiation (28). *Il12rb2* was also repressed by over expression of Tox/Tox2 in CD8 and CD4 T cells and correlated with their reduced capacity to produce IFN-γ (6, 11), indicating that this is a function of Tox in both T cell types.

Tox induced and repressed a more refined set of genes in unpolarized Th cells as compared to CD8 T cells under similar settings. Here we showed that over expression of Tox significantly modulated (>2-fold induction or repression) a relatively small set of 22 genes in activated CD4 T cells as compared to CD8 T cells where hundreds of genes were induced or repressed to the same criteria (6). In previous work, Xu et al. also noted that Tox transduction in similar CD4 T cell conditions induced expression of a number of TFH-associated genes (*Bcl6, Ascl2, Cxcr5*) that we did not identify in our study. A key difference between our study and that of Xu et al. is that we added recombinant IL-2 to our cultures, where IL-2 was either not added or blocked in this previous work. Interestingly, the majority of genes identified in Xu et al. are repressed by IL-2. Therefore, these differences in culture conditions (i.e. +/- IL-2) is likely to explain these differences. Of the Tox-modulated genes we identified, 4 of these were similarly regulated in CD8 T cells (*Pdcd1, Ccl3, Asb2, Il12rb2*). Interestingly, 3 of the 22 genes we identified (*Xcl1, Ccl3, Ccl4*) were involved in facilitating T cell-myeloid cell interactions and in the recruitment of immune cells to sites of inflammation. The XCL1 receptor, XCR1, is almost exclusively expressed on dendritic cells and CCL3 and CCL4 enhance the recruitment of CCR5^+^ cells (myeloid cells, T cells) to inflamed sites. Previous work also indicates that CD4 T cell-derived CCL3/CCL4 act to recruit CD8 T cells to the site of active dendritic cell-CD4 T cell interactions in secondary lymphoid organs (32) like the Peyer’s Patch. As XCL1 also induces the recruitment of dendritic cells (33), these data implicate Tox as a factor that orchestrates the three-way interaction that leads to dendritic cells licensing and effective induction of the CD8 T cell response.

We demonstrated that the anti-inflammatory cytokine IL-10 was also elevated at the mRNA and protein level in both Tox RV-transduced CD4 and CD8 T cells. This is of particular importance as IL-10 production by T cells is enhanced and correlates with heightened T cell Tox expression in chronic viral infection (6, 34, 35). Blockade of IL-10 resolved chronic viral infection and T cell exhaustion in some settings (35), suggesting that Tox-mediated IL-10 production may play a negative role in resolving infection. In the setting of cancer biology, tumor-infiltrating CD4 and CD8 T cells also express elevated Tox and have been shown to produce IL-10 (4, 36, 37). IL-10 production in tumors has been shown to have potential beneficial effects in promoting T cell effector function (i.e. IFN-γ and granzyme production) and limiting tumor growth (36–40). Our data therefore suggest a mechanism by which Tox-induced IL-10 production by tumor-infiltrating T cells and may act to maintain T cell effector activity. In line with this hypothesis, Tox-deficient CD8 T cells exhibited reduced cytokine production, killing capacity and tumor persistence despite having diminished expression of inhibitory surface molecules (PD-1, LAG3) (4). Further *in vivo* work must be done to determine the individual role of Tox-induced IL-10 on these phenomena.

Similar to Tox, the basic leucine zipper transcription factor BATF is also involved in TFH differentiation (41, 42), T cell IL-10 production (43) and the exhaustion of chimeric antigen-receptor T cells in tumors (44). These data suggest that Tox and BATF may cooperate to regulate these distinct processes. Indeed, recent data from terminally exhausted CD8 T cells suggests that the expression of Tox and BATF are upregulated and that these factors co-occupy a high frequency of genomic regions that are associated with T cell exhaustion (16). In a metanalysis of these data and others, we show that Tox along with BATF, IRF4 and JunB co-occupy in CD8 T cells and these sites correspond to BATF/IRF4/JunB-bound sites in CD4 T cells. In line with these data, we show that BATF was indeed required for Tox-induced IL-10 production (**Figure 4**). Despite these factors also co-occupying several sites in and around the Pdcd1 locus in CD8 T cells, this was not conserved in cultured CD4 T cells (data not shown). Further, we found that Tox-induced PD-1 expression was BATF-independent for both cell types. These data suggest that Tox alone or Tox interactions with IRF4 or JunB alone are sufficient to drive expression of PD-1. Further work must be done to characterize Tox-induced gene loci that are BATF-dependent or-independent to answer this question. Examining these phenomena in CD8 T cells, where there are a large number of Tox-induced genes, is likely a better system for these analyses. Taken together however, our data lays an important framework that Tox regulates transcription of its target genes through both BATF-dependent (i.e. *Il10*) and -independent (i.e. *Pdcd1*) mechanisms.

As a whole, our data indicate that Tox is expressed by CD4 T cells in healthy peripheral tissues and that these non-chronically activated/non-TFH cells may have Tox-dependent functions within these locales. As Tox is associated with T cell exhaustion in tumors, it has become a potential target for therapeutic targeting. However, because Tox^+^ CD4 T cells in peripheral/mucosal tissues likely play immunomodulatory roles, disruption of their function by Tox inhibitors may result in increased immune-related adverse effects. Finally, factors that bolster Tox expression by these peripheral CD4 T cells may improve their ability to aid in induction of *de novo* T cell responses by facilitating dendritic cell-T cell interactions and potentially regulating unwanted inflammatory responses through the production of IL-10.

Our study has some limitations that need to be considered. We propose that Tox functions with a TCR-induced factor (BATF) to enhance the production of IL-10 under acute TCR stimulation settings. However, other Tox-induced functions may only be apparent under settings of chronic TCR activation (i.e. sustained Tox and BATF expression) that might be missed in our study with only acute TCR stimulation. In a similar fashion, our studies to this point have been solely performed *in vitro*. While this allows us to take a very reductionist/mechanistic approach to how specific genes are regulated, these findings must be validated *in vivo* during anti-tumor or anti-viral immune responses where Tox is highly expressed by T cells. Finally, we waited two days post-onset of culture (e.g. initial TCR stimulation) to transduce T cells with Tox. As BATF is induced rapidly after TCR stimulus (~24 hours), we may miss important early Tox-BATF interactions that drive epigenetic programming at this state that may act to have robust changes in cellular function. Despite these limitations, we have laid an important initial framework for future studies examining early events after TCR signaling and how these Tox-induced genes contribute to *in vivo* immune responses to cancer and chronic infection.

## Data availability statement

The datasets presented in this study can be found in online repositories. The names of the repository/repositories and accession number(s) can be found here: GSE240348 (GEO).

## Ethics statement

The animal study was approved by Purdue University Institutional Animal Care and Use Committee. The study was conducted in accordance with the local legislation and institutional requirements.

## Author contributions

DC: Investigation, Writing – original draft, Writing – review & editing, Data curation, Formal Analysis, Methodology. JR: Data curation, Formal Analysis, Investigation, Writing – review & editing. LW: Data curation, Formal Analysis, Writing – review & editing. FY: Data curation, Formal analysis, Writing – review & editing. BY: Data curation, Formal Analysis, Writing – original draft. MW: Formal Analysis, Writing – review & editing, Data curation. CC: Formal Analysis, Writing – review & editing, Investigation. MK: Conceptualization, Data curation, Formal Analysis, Writing – review & editing. MO: Conceptualization, Writing – review & editing, Funding acquisition, Investigation, Project administration, Resources, Supervision, Writing – original draft.
